# Nanobiotechnology

**DOI:** 10.1155/2013/319832

**Published:** 2013-09-16

**Authors:** Susana N. Diniz, Alejandro Sosnik, Huiling Mu, Claudete J. Valduga

**Affiliations:** ^1^Bandeirante Anhanguera University, 02071-013 Sao Paulo, SP, Brazil; ^2^University of Buenos Aires, 1113 Buenos Aires, Argentina; ^3^University of Copenhagen, 2100 Copenhagen, Denmark

Nanobiotechnology refers to methods and techniques inspired by biology to develop products at the nanometer scale. This modern concept describes the way nanotechnology is used to create devices in order to study biological systems. It is based on the overlapping of multidisciplinary sciences converging to create novel nanostructures that are similar to nature to sense and deliver biomacromolecules. This special issue is compiled with seven articles that explore new approaches on the development of products to address medical/biological problems for diagnosing and treating diseases. Two of them are reviews about the application of nanoparticles in drug delivery and diagnosis.

Biocompatible and biodegradable materials have great potential in nanobiotechnology. The review by A. Shrivastav et al. focuses on nanoparticles based on natural occurring polymeric material, polyhydroxyalkanoates, and their applications in drug delivery. 

Diagnosis and treatment of some kinds of cancers can be difficult such as gliomas, which show high resistance to chemotherapy and radiotherapy. The potential of nanobiotechnology for drug delivery, imaging, diagnosis, and therapy was addressed in the review by N. Y. Hernández-Pedro et al.

The development of nanoscale tools for understanding the mechanisms of cellular functions and monitoring the behavior of biomolecules, cell surface interactions, as well as new approaches in drug and gene delivery was the focus of five articles.

The penetration of antineoplastic drugs into solid tumors is a hurdle that can be bypassed by the use of nanodevices, as showed by Y. Liu et al., in which a crosslinked multilamellar liposomal vesicle formulation with a tumor-penetrating peptide, iRGD, is able to improve the delivery of doxorubicin to breast tumor cells *in vitro* and *in vivo*. 

D. Shahbazi-Gahrouei et al. illustrate the potential of magnetic nanoparticles for detection and quantification of cell surface antigens in prostate cancer cells. The transfection of RNA by L. Russoa et al., antisense oligonucleotide by J. Xie et al., and plasmid DNA by L. Prossen et al. show the potential of nanobiotechnology for gene therapy and control of antiproliferative diseases.

We are certain that the articles published in this special issue will contribute to the development and application of nanobiotechnology especially as a biological tool for diagnostics and the treatment of diseases.

*Susana N. Diniz* *Alejandro Sosnik* *Huiling Mu* *Claudete J. Valduga*

